# Poisoning among Autopsies Conducted in the Department of Forensic Medicine and Toxicology in a Tertiary Care Centre

**DOI:** 10.31729/jnma.8142

**Published:** 2023-08-31

**Authors:** Abdul Sami Khan, Archana Pandey, Ajit Pandey

**Affiliations:** 1 Department of Forensic Medicine and Toxicology, Kathmandu University School of Medical Sciences, Dhulikhel, Kavrepalanchowk, Nepal; 2 Kathmandu University School of Medical Sciences, Dhulikhel, Kavrepalanchowk, Nepal

**Keywords:** *autopsy*, *organophosphate poisoning*, *poisoning*, *suicide*

## Abstract

**Introduction:**

Poisoning is a serious public health issue in developing countries like Nepal. Information about poisoning may be helpful for poisoning prevention and hospital treatment, aiding in the development of measures that lower the morbidity and mortality associated with poisoning. This study aimed to find out the prevalence of poisoning among autopsies conducted in the Department of Forensic Medicine and Toxicology in a tertiary care centre.

**Methods:**

A descriptive cross-sectional study was conducted among autopsied cases in the Department of Forensic Medicine and Toxicology in a tertiary care centre. Data from 1 October 2020 to 1 April 2022 was collected between 22 December 2022 to 30 December 2022 from records after receiving ethical approval from the Institutional Review Committee. All autopsied cases during the study period were included with the exclusion of decomposed bodies. Convenience sampling method was used. The point estimate was calculated at a 95% Confidence Interval.

**Results:**

Among 399 autopsies, 63 (15.79%) (12.21-19.37, 95% Confidence Interval) were found to be cases of poisoning. Among 63 cases, 35 (55.56%) were male and 28 (44.44%) were female. The most common substance causing poisoning was unknown with 31 (49.21%) cases, followed by organophosphates with 24 (38.10%) cases and rodenticide with 8 (12.70%) cases.

**Conclusions:**

The prevalence of poisoning among autopsies was found to be higher than similar studies conducted in similar settings.

## INTRODUCTION

Nepal had a suicide rate of 9 per 100,000 population in 2019.^1^ In the same year, over 700, 000 people died by suicide worldwide and the number of deaths from unintentional poisoning was approximately 84,000, or 1.1 per 100,000.^2^ Pesticide poisoning is the most prevalent means of suicide, in low and middle-income nations.^3^

Poisoning is a major issue in Nepal since it is predominantly an agricultural nation and organophosphates are widely used in the area, they are easily accessible and may be purchased over the counter at retail stores in Nepal.^4^ Information about poisoning may be useful in both preventing and treating such cases in hospitals.

This study aimed to find out the prevalence of poisoning among autopsies conducted in the Department of Forensic Medicine and Toxicology in a tertiary care centre.

## METHODS

A descriptive cross-sectional study was conducted among autopsies done at the Department of Forensic Medicine and Toxicology, Kathmandu University School of Medical Sciences, Dhulikhel, Kavrepalanchowk, Nepal. Data from 1 October 2020 to 1 April 2022 was collected between 22 December 2022 to 30 December 2022 from records after receiving ethical approval from the Institutional Review Committee. after obtaining ethical approval from the Institutional Review Committe (Reference number: 243/22). All autopsied cases during the study period were included while decomposed bodies were excluded from the study. Convenience sampling was done. The sample size was calculated by using the following formula:


$$\begin{array}{ll} \text{n} & ={\text{Z}}^{2}\times \frac{\text{p}\times \text{q}}{{\text{e}}^{\text{2}}}\\ & =1.96^{2}\times \frac{0.11\times 0.89}{0.08^{2}}\\ & =59 \end{array}$$

Where,

n = minimum required sample size
Z = 1.96 at 95% Confidence Interval (CI)
p = prevalence taken from a previous study, 11.48%^5^
q = 1-p
e = margin of error, 8%

The minimum required sample size was 59. However, the final sample size taken was 63. Detailed information regarding the circumstances of the death was collected from the inquest, hospital records and medico-legal autopsy register. Data was compiled and analysed as per age, sex, place of residence, calendar month, type of poison, the time interval between ingestion of poison and death, treatment, stomach content, congestion of viscera, manner of death, route of exposure using a predesigned proforma.

Data were entered and analyzed using IBM SPSS Statistics version 21.0. The point estimate was calculated at a 95% Confidence Interval.

## RESULTS

**Figure 1:**
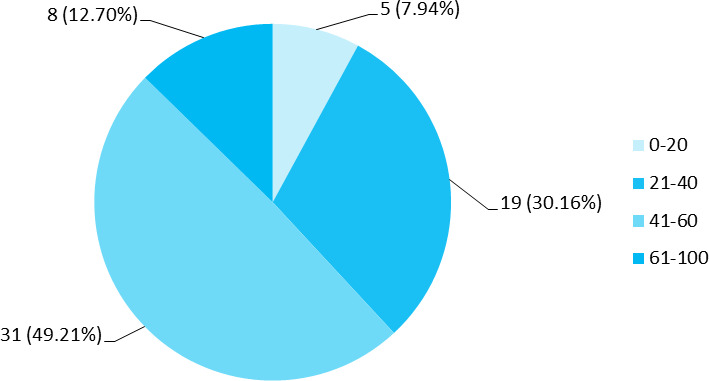
Age-wise distribution of cases (n= 63).

Among 399 medico-legal autopsies, 63 (15.79%) (12.2119.37, 95% CI) were found to be cases of poisoning out of which 35 (55.56%) were male and 28 (44.44%) were female. Thirty-one (49.21%) of poisoning cases were in the age group of 41-60 years (Figure 1).

The majority of cases were from rural locality with 55 (87.30%) cases and 8 (12.70%) cases being from urban locality. Out of 63 poisoning cases, the maximum number of deaths occurred due to unknown poisons in 31 (49.21%) cases followed by organophosphorus compounds in 24 (38.10%) (Table 1).

**Table 1:** Type of poison consumed (n= 63).

Type of poison	n (%)
Organophosphorus	24 (38.10)
Rodenticide	8 (12.70)
Unknown	31 (49.21)

**Figure 2:**
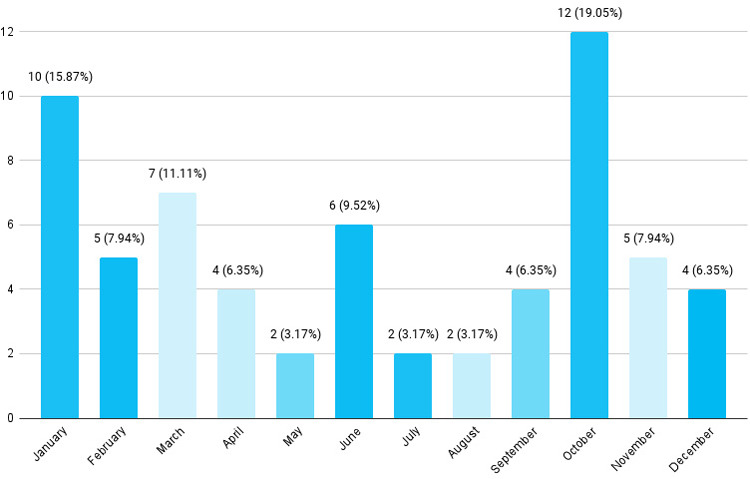
Month-wise distribution (n= 63).

Out of 63 poisoning cases, the maximum number of deaths occurred in the month of October in 12 (19.05%) followed by January with 10 (18.87%) cases (Figure 2).

**Figure 3:**
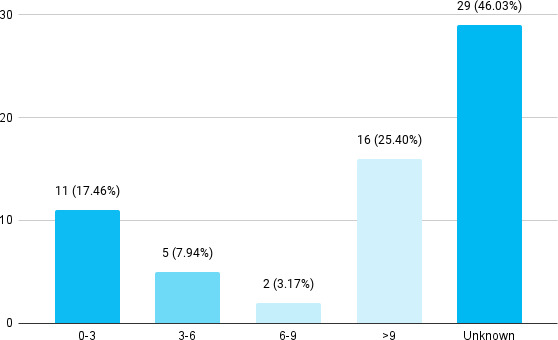
Time interval between ingestion and death (n= 63).

Among 63 poisoning cases, 16 (25.40%) died after 9 hours (Figure 3).

The stomach content of the maximum number of cases had a kerosene smell in 33 (52.38%) cases, garlic smell in 5 (7.94%) and no smell in 25 (39.68%) cases. Out of 63 poisoning cases autopsied, 54 (85.71%) cases were due to suicide (Table 2).

**Table 2:** Manner of death (n= 63).

Manner of Death	n (%)
Suicidal	54 (85.71)
Homicidal	2 (3.17)
Accidental	1 (1.59)
Unknown	6 (9.52)

A majority of these cases 32 (51.79%) did not receive treatment. Among 63 poisoning cases, 49 (77.78%) cases had congested viscera.

## DISCUSSION

In this study, out of 399 autopsies performed, the prevalence of poisoning was found to be 63 (15.78%). This finding was similar to a study done in a similar setting where the prevalence of poisoning was found to be 11.48%.^5^

According to the findings, males 35 (55.56%) were impacted more often than females which is similar to various studies.^2,6^ This result may be supported by a study that revealed men reportedly died by suicide more than women in all age groups.^7^ In this study, the maximum number of deaths occurred due to unknown poison 31 (49.21%) followed by organophosphorus compounds 24 (38.10%) and rodenticide 8 (12.70%). Easy availability of pesticides is the primary cause of this increased rate of poisoning in Nepal with 18,000 fold higher rate of OP poisoning in comparison to the US.^8^ Organophosphates have been the most used poisons in multiple studies in Nepal.^9,10^ The highest number of fatalities due to poisoning 12 (19.05%) occurred in October, the period of the year when pesticides are used most often. Previously methyl parathion was one of the most common pesticides involved in poisoning, before it was banned in 2007.^11^

165 pesticides are currently registered in the nation. One (0.6%) is very dangerous (WHO class Ia), one (0.6%) is highly hazardous (WHO class Ib), 70 (42.42%) are moderately harmful (WHO class II), 26 (15.75%) are somewhat hazardous (WHO class III), two are unclassified, and 65 are unlikely to provide an acute threat (WHO class U).^11^ The Food and Agriculture Organization created the Farmer Field Schools (FFS) strategy in South East Asia in the late 1980s to help farmers acquire the knowledge and advantages of using Integrated Pest Management (IPM) in their crops.^12^ Studies on this strategy showed a decrease in pesticide poisoning instances or poisoning signs and symptoms and were linked to lower pesticide applications, the use of less hazardous products, or the use of enhanced personal protection techniques.^13^

Poisoning was the second most common method of suicide between 2015 to 2019.^14^ However, the exact number of suicide fatalities may be greater than estimated since suicides in rural regions are seldom recorded due to social stigma as well as the false belief that suicide is against the law in Nepal. Another study involving poisoning in hospitalized patients found that the highest percentage of poisoning was related to deliberate self-harm, which was similar to our case, but it was followed by depression and accidental poisoning in the other study, whereas suicide was followed by undetermined poisoning and homicidal poisoning in our case.^8^ Only 1 case of accidental poisoning shows that the pesticides presently being used in Nepal after the prior bans only occasionally cause serious occupational poisoning.

This study found that 8 cases (12.70%) were from urban regions, compared to 55 cases (87.30%) from rural areas. Agriculture engagement, a lack of rural health care services, and the accessibility of agrochemicals may be too accountable. Many fatalities occurring outside of or near peripheral institutions may be a result of inadequate access to healthcare, a lack of high-quality healthcare in the periphery, or a result of rapid onset of action of poisons, which is consistent with other studies which claim fatality depends on access to quality healthcare.^15^

The data might not accurately reflect the challenge facing the broader public because they were only acquired from health facilities. As previously stated, the actual number of poisoning fatalities may be far higher than what we discovered because many instances remain unreported.

## CONCLUSIONS

The prevalence of poisoning among autopsied cases was found to be higher than other studies performed in similar settings. Suicide prevention should also be addressed in Nepal. The need for strict pesticide usage and procurement rules is crucial to reduce deaths.
